# Pro Perspective

**DOI:** 10.1192/j.eurpsy.2022.192

**Published:** 2022-09-01

**Authors:** P. Gorwood, L. Di Lodovico

**Affiliations:** 1 University of Paris, Cmme, Hopital Sainte-anne Ghu Paris Psychiatrie Et Neurosciences, Paris, France; 2 GHU Paris Psychiatrie, Clinique Des Maladies Mentales Et De L’encéphale, Paris, France

## Abstract

Four sets of arguments are supporting the idea that compulsory admissions should be possible in anorexia nervosa, as much as it is other in psychiatric disorders. Indeed, if nobody challenges the use of compulsory treatments for patients with acutte schizophrenia or a manic episodes, it is because the usual rational for compulsory admission relies on (1) the severity of the disorder (a need), (2) the capacity of hospitalisation to really improve patients and prognosis (a utility), associated to the fact that (3) the disorder alters the capacity to spontaneously ask for care (a rational), and (4) the possible danger of the present medical conditions for others (a protection). We will explain that these four points are clearly being equivalent for anorexia nervosa compared to other psychiatric disorders, with some subtilities that modify their expression, but not the spirit of these rationals. We will then propose that a much easier way to understand the paicity of use of this hospitalisation modalities is related to the strange position of anorexia nervosa, a metabo-psychiatric disorder, namely a complex disorder at the interface of somatic and psychiatric disorder. We will conclude in promoting a step-by-step procedure to reuce the risk of abuse, and facilitate the paradoxical “acceptance” of a “forced” hospitalisation.

**Disclosure:**

No significant relationships.

